# An expanded method for malaria parasite genetic surveillance using targeted nanopore sequencing

**DOI:** 10.12688/gatesopenres.16355.1

**Published:** 2025-07-24

**Authors:** Alexandria J. R. Harrott, Collins M. Morang'a, Richard D. Pearson, Mona-Liza Sakyi, Ahmed Osumanu, Enock K. Amoako, Fagdéba David Bara, Myra Hosmillo, Kess Rowe, Yaw Aniweh, Gordon A. Awandare, Francis Zeukeng, Ian Goodfellow, Cristina V. Ariani, Lucas N. Amenga-Etego, William L. Hamilton

**Affiliations:** 1 Wellcome Sanger Institute, Hinxton, England, CB10 1RQ, UK; 2 University of Bath Department of Life Sciences, Bath, England, BA2 7AZ, UK; 3 West African Centre for Cell Biology of Infectious Pathogens, Legon, Accra, Ghana; 4 University of Cambridge Department of Pathology, Cambridge, England, CB2 0QQ, UK; 5 University of Yaoundé 1, Etetak-Yaounde, P.O. box 17673, Cameroon; 6 University of Buea, Buea, P.O. box 63 Buea, Cameroon; 7 University of Cambridge Department of Medicine, Cambridge, England, CB2 0QQ, UK; 8 Cambridge University Hospitals NHS Foundation Trust, Cambridge, England, CB2 0QQ, UK

**Keywords:** Malaria, nanopore, P. falciparum, surveillance, antimicrobial resistance, AMR, vaccine, amplicon sequencing

## Abstract

Malaria causes around 250 million cases and over 600,000 deaths annually, with the heaviest burden falling on young children living in sub-Saharan Africa. Molecular surveillance of *Plasmodium* parasites and *Anopheles* mosquito vectors are key components of effective malaria control decision-making. Previously, we have designed and implemented a nanopore-based workflow for targeted *P. falciparum* molecular surveillance in Ghana, which we call DRAG1 (drug resistance + antigen multiplex PCR). Here, we describe an updated and expanded multiplex assay (‘DRAG2’) with additional amplicon targets that incorporate more antimalarial drug resistance markers, the polymorphic surface antigen *merozoite surface protein 2* ( *msp2*), and the 18S ribosomal RNA (rRNA) gene for *Plasmodium* species detection. We describe the performance of the DRAG2 assay over a range of parasitaemias and sample types (venous blood and dried blood spots), with suggested systems of quality control including the use of synthetic plasmids for positive controls and recommended coverage thresholds. The plasmids are highly economical, and engineered to include both ‘test’ single nucleotide polymorphisms (SNPs), such as known drug resistance markers, and ‘control’ SNPs, which are not found in nature and thus signal contamination if detected in clinical samples. We provide standard operating procedures (SOPs) for use by teams aiming to implement the assay in their laboratory. In summary, we describe an updated nanopore-based method for malaria molecular surveillance, including detailed consideration of quality control processes and SOPs. These are important steps in the transition from research tool to diagnostic assay, which will require further testing in endemic settings and regulatory processes and approvals.

## Plain language summary

Malaria is a major cause of disease and death globally, especially for young children living in Africa. Affected countries aim to reduce the spread of the malaria parasite, which is transmitted by mosquitoes, and to treat infected people using drugs. However, the parasites can develop resistance to the drugs, making them less effective. Newly approved malaria vaccines are also being deployed at scale. It is therefore important to monitor parasites for the development and spread of drug resistance and for any changes relevant to the vaccine, to ensure treatment outcomes remain optimal. This study describes a laboratory method for monitoring malaria parasites suitable for use in tropical areas where malaria is common. Starting from a blood sample taken from an infected person, targeted sections of malaria parasite DNA are amplified and sequenced using nanopore technology. The genes analysed provide information on drug resistance, the current vaccine target, and which malaria species are present. The study also describes laboratory practices to ensure high quality standards are maintained consistently. In summary, this study reports a method for monitoring malaria parasites using a streamlined DNA sequencing workflow; further work can assess its performance in malaria zones and develop protocols suitable for clinical use.

## Introduction

Malaria exacts a huge toll on human health globally, particularly for young children living in sub-Saharan Africa. There were 249 million malaria cases and 608,000 deaths in 2022 across 85 countries. ^1^ The number of cases in 2022 increased by five million compared to 2021. 95% of cases and deaths were in sub-Saharan Africa, and around four in five malaria deaths were children under five years old. Threats to malaria control include antimalarial drug resistance, deletions in the diagnostic target genes *hrp2* and *hrp3*, vector insecticide resistance, and the spread of invasive vector species. Continuous political will and investment to sustain and intensify control efforts is essential. A changing climate may further contribute to fluctuations in transmission dynamics in the coming years.

Despite these challenges, there are multiple new interventions being added to the antimalarial armamentarium, including novel antimalarial drugs, long-acting monoclonal antibodies, the expansion of molecular systems of surveillance in endemic countries, and several vaccines. Currently, two malaria vaccines are recommended by the World Health Organization (WHO), RTS, S and R21, which both target circumsporozoite protein (PfCSP) in the most virulent malaria parasite species, *Plasmodium falciparum.* Parasite populations are therefore being placed under a multitude of intense selection pressures, creating the ecological conditions that may drive the evolution of antimalarial drug resistance or possibly vaccine escape mutants. In this dynamic context, continuous surveillance of parasite populations forms a critical component of malaria control, allowing national malaria control programmes (NMCPs) to deploy their resources most effectively and respond quickly to heritable changes in the parasite that confer fitness against interventions. ^2^

Nanopore sequencing has been increasingly used to generate genetic data on *Plasmodium* parasites, including in endemic countries, such as detecting antimalarial drug resistance markers, variation in the *csp* gene sequence, *hrp2/3* gene deletions, and population dynamics. ^3– 6^ Nanopore technology offers advantages including relatively low up-front costs, ‘real-time’ sequencing, portability, scalability, and long sequence reads. Moreover, use of Oxford Nanopore Technologies (ONT) has expanded in malaria endemic countries during the Covid-19 pandemic, in efforts to increase global SARS-CoV-2 genomic surveillance. ^7^ However, significant challenges to implementation persist, including optimising sampling strategy, the low quality and quantity of parasite DNA generally retrieved from clinical samples, slow procurement processes in many endemic countries, and bioinformatics capacity (reviewed in Ref. 8).

Previously, we have developed and implemented a nanopore-based multiplex amplicon assay for *P. falciparum* genomic surveillance in Ghana. ^5^ This assay had targets within six genes, five for monitoring antimalarial drug resistance ( *chloroquine resistance transporter* ( *crt*), *dihydrofolate reductase* ( *dhfr*), *dihydropteroate synthase* ( *dhps*), *multidrug resistance protein 1* ( *mdr1*), and *kelch13*), and one for vaccine target surveillance ( *csp*). We refer to this assay as ‘DRAG1’ (drug resistance + antigen multiplex PCR). Here, we expand on this work by adding additional targets to the multiplex PCR, producing a new assay we refer to as ‘DRAG2’. We describe assay performance, quality control processes including coverage thresholds and use of synthetic plasmids as positive controls, and standard operating procedures (SOPs) for assay implementation.

## Methods

### DRAG2: An expanded multiplex PCR for malaria surveillance using nanopore sequencing

The DRAG2 multiplex PCR builds on our previous iteration of the assay (‘DRAG1’) by adding additional amplicons for the drug resistance genes *crt* and *mdr1*, the full-length *merozoite surface protein 2* ( *msp2*) gene, and a target within the small subunit of ribosomal rRNA genes that can distinguish between *Plasmodium* species. A summary of the amplicons is shown in Table 1 and Extended data (all supplementary materials for this project are freely available at: https://doi.org/10.6084/m9.figshare.28539320.v1). ^27^ We divided the assay into two separate multiplex reactions, which we refer to as DRAG2-A and DRAG2-B. This reduced the risk of primer interactions and increased target specificity. In addition to the drug resistance markers targeted in DRAG1, the expanded DRAG2 assay includes C-terminal sections of *crt* and *mdr1* ( Table 2). Full-length *msp2* was included due to its polymorphism, with potential to act as an approximate indicator of multiplicity of infection (MOI) i.e. infections with multiple *P. falciparum* clones. While the assay is intended as a tool for *P. falciparum* molecular surveillance, the 18S rRNA gene target was included to detect mixed species infections or unintended non- *falciparum* malaria samples; further details are provided below. The amplicon targets were designed to be more similar to each-other in size compared with DRAG1, to make sequencing coverage more even (average and standard deviation (SD) of amplicon sizes in the 3D7 reference genome for DRAG1 and DRAG2 assays are 586bp (SD 303bp) vs. 668bp (SD 166bp), respectively).

**Table 1.** Primer sequences and amplicon targets included in the DRAG2 assay. The assay is divided into two multiplex PCR mixtures, DRAG2-A and DRAG2-B. Amplicon size is relative to the 3D7 reference genome. ‘New’ refers to whether the primer sequences are new since the DRAG1 assay (already published), or were already included in DRAG1. Primer sequences are also shown in Supplementary Table 2.

DRAG2 assay	Primer name	Gene target	Amplicon type	Primer sequence	Amplicon size (bp)	New from DRAG1
A	kelch13 - F	kelch13	Drug resistance	AAGCCTTGTTGAAAGAAGCA	868	No
	kelch13 - R	kelch13	Drug resistance	GGGAACTAATAAAGATGGGCC		No
	18S rRNA - F	18S rRNA	Species detection	CAATTGGAGGGCAAGTCTG	690 & 748	Yes
	18S rRNA - R	18S rRNA	Species detection	CTTTTAACTTTCTCGCTTGCG		Yes
	crt-C - F	crt	Drug resistance	ACCTTCGCATTGTTTTCCTTC	530	Yes
	crt-C - R	crt	Drug resistance	AGTTACGAAATCTAATAATCTTGGTTC		Yes
	dhfr - F	dhfr	Drug resistance	GTTTTCGATATTTATGCCATATGTG	490	No
	dhfr - R	dhfr	Drug resistance	TGATAAACAACGGAACCTCC		No
	mdr1-N - F	mdr1	Drug resistance	CCGTTTAAATGTTTACCTGCAC	459	Yes
	mdr1-N - R	mdr1	Drug resistance	ACATAAAGTCAAACGTGCATTTT		Yes
B	csp - F	csp	Antigen	TGGGAAACAGGAAAATTGGTAT	975	No
	csp - R	csp	Antigen	TACGACATTAAACACACTGGAA		No
	msp2 - F	msp2	Antigen	TGAAAGTAAATATAGCAACACATTCAT	750	Yes
	msp2 - R	msp2	Antigen	ATATGGCAAAAGATAAAACAAGTGT		Yes
	mdr1-C - F	mdr1	Drug resistance	TGTAAATGCAGCTTTATGGGG	703	Yes
	mdr1-C - R	mdr1	Drug resistance	CATGGGTTCTTGACTAACTATTGA		Yes
	dhps - F	dhps	Drug resistance	TTTGTTGAACCTAAACGTGC	641	No
	dhps - R	dhps	Drug resistance	AACATTTTGATCATTCATGCAAT		No
	crt-N - F	crt	Drug resistance	TGGAGGTTCTTGTCTTGGTA	494	Yes
	crt-N - R	crt	Drug resistance	ACTGAACAGGCATCTAACATG		Yes

**Table 2.** Specific drug resistance marker targets included in DRAG2 assay. Red text indicates targets added in DRAG2 that are not included in DRAG1. Both DRAG1 and DRAG2 assays also include the region of *kelch13* in which artemisinin resistance mutations have been identified and the vaccine target *csp.*

Gene name and ID in 3D7 reference	Key mutations targeted for genotyping	Associated antimalarial resistance
Chloroquine resistance transporter, *crt* (PF3D7_0709000)	N-terminal: K76T C-terminal: N326S, I356T	Chloroquine resistance marker
Dihydrofolate reductase, *dhfr* (PF3D7_0417200)	N51I, C59R, S108N, I164L	Pyrimethamine resistance markers
Dihydropteroate synthase, *dhps* (PF3D7_0810800)	S436A, A437G, K540E, A581G, A613S/T	Sulfadoxine resistance markers
Multidrug resistance protein 1, *mdr1* (PF3D7_0523000)	N-terminal: N86Y, N86F, Y184F C-terminal: S1034C, N1042D, D1246Y	No direct inferences, but associated with resistance to several antimalarials including lumefantrine

### Primer design for multiplex PCR

Primer design followed a similar process to, ^5^ using primer3 software ^9– 11^ to generate candidate primer sequences, followed by *in silico* checks for primer interactions with ThermoFisher Multiple Primer Analyzer, ^12^ and *in vitro* testing of primer combinations to identify high-performing combinations based on PCR product inspection by agarose gel electrophoresis and nanopore sequencing to determine gene target coverage profiles.

For species detection, we chose the 18S rRNA gene because (1) this is already well described as a target for *Plasmodium* species detection ^13– 15^; (2) 16S and18S gene amplicon sequencing is already well used and well understood in clinical microbiology practice as a molecular method of microbial identification ^16^; (3) the multiple copies of 18S rRNA in the *Plasmodium* genome effectively increases the amount of template DNA present for amplification, which may increase sensitivity; (4) we found improved multiplex PCR performance if all primers targeted the nuclear genome, rather than adding mitochondrial targets for species detection to the same PCR reaction as nuclear genome targets, perhaps due to different AT-content and extremely different ploidy between the mitochondrial and nuclear genomes. 16S and 18S rRNA genes have sections of high sequence conservation interspersed with regions that vary between species, making them well suited for microbial identification via primers that target the more conserved regions. This approach is also well suited to the long sequence reads possible with ONT.

Primer sequences (except for the 18S rRNA primers) were selected in conserved regions across high-quality *P. falciparum* genomes spanning wide geographic origins ^17^ based on multiple sequence alignments, and then screened for variants in the Pf7 MalariaGEN *P. falciparum* Community Project data resource. ^18^ For 18S rRNA primer design, publicly available 18S rRNA sequences from *P. falciparum*, *P. vivax*, *P. ovale-curtisi *, *P. ovale-wallikeri *, and *P. knowlesi* were aligned to identify conserved regions between all species for primer placement, containing regions that varied between species within the amplified sequence. The 18S rRNA primer pair chosen was not expected to amplify a product from the human genome (this was subsequently confirmed using a human gDNA sample).

### Use of plasmid mixtures as positive controls

Positive and negative controls are essential components of quality assurance in diagnostic microbiology. Negative controls should undergo the same end-to-end processes as test samples, including using the same reagents. This should identify reagent contamination, which could lead to false inferences by assigning contaminant genotypes to clinical samples (contamination risk is reduced by strict separation of pre- and post- PCR laboratory locations, pipettes and tips). Positive controls are required (1) to identify where problems are arising in a laboratory protocol; and (2) to confirm that the assay should have been able to detect a true positive (for example, if all test samples are negative). Positive control material could also form part of internal and external quality assurance processes; for example, by testing samples with known resistance genotypes and ensuring concordance, and investigating any discrepancies. Using *P. falciparum* genomic DNA as positive controls has several disadvantages, including: (1) a risk of clinical sample contamination, leading to false conclusions; (2) it can be slow and resource-intensive to culture *P. falciparum* parasites *in vitro* to generate gDNA material, requiring human erythrocytes and laboratory equipment; (3) it may be difficult or impossible to obtain parasites and/or genetic material that contain specific mutations to test assay performance, such as *kelch13* mutations found in non-culture adapted parasite lines; (4) non- *falciparum* malaria species are difficult to culture and obtain genetic material, making positive controls for *Plasmodium* species identification challenging.

We addressed these challenges by designing a system of synthetic plasmids for use as positive controls. The plasmids were purchased from a commercial company (GenScript) using a pUC57 vector background. Each plasmid contained a *Plasmodium* gene section targeted in the DRAG1 and DRAG2 PCRs, including the non- *falciparum* 18S rRNA gene sequences Extended data; Supplementary Tables 3-4). ^27^ Each plasmid insert sequence was based on the 3D7 reference genome (or the corresponding non- *falciparum* reference genomes for 18S rRNA genes), but modified in two ways: (1) ‘test SNPs’ were added, consisting of known drug resistance mutations or other genetic changes the assay should detect; and (2) ‘control SNPs’, which were SNPs never found in nature (based on screening the Pf7 MalariaGEN data resource) and which result in amino acid substitutions with BLOSUM62 scores of -2 or below, suggesting they are biologically unlikely. Full details of test and control SNPs for the plasmid inserts are shown in Extended data; Supplementary Table 5), ^27^ and the fasta sequences are provided in Extended data. ^27^ At least two test SNPs and two control SNPs were added per insert sequence. It is extremely unlikely that two control SNPs would genuinely occur in a clinical sample; therefore, these SNPs act as markers of positive control contamination in clinical samples, and would facilitate contamination being quantified highly accurately from sequence read counts. Quality assurance systems could monitor for control contamination levels, with cut-offs to trigger investigation based on absolute read counts, relative read counts, and trends over time.

**Figure 1.**
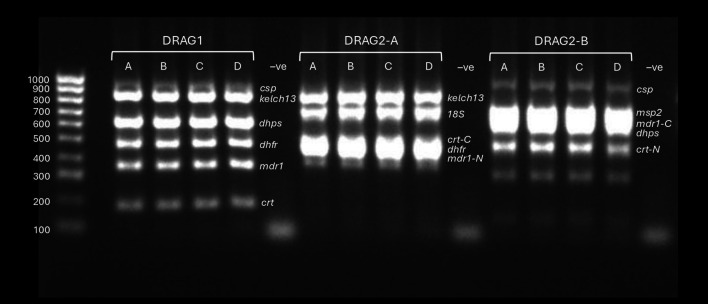
Agarose gel electrophoresis of DRAG1 and DRAG2 multiplex PCRs. DRAG1, DRAG2-A and DRAG2-B multiplex PCRs visualised by 2% agarose gel electrophoresis. Samples A-C use plasmid mixtures containing the inserts targeted in the assays at different concentrations; sample D is *P. falciparum* genomic DNA (laboratory clone DD2). The plasmid mixtures for samples A-C were, respectively: Mixture 1 containing the drug resistance and *csp* target regions plus three *P. falciparum* 18S rRNA sequences, Mixture 2 with added *P. malariae* 18S rRNA sequences *,* and Mixture 3 with added *P. ovale* 18S rRNA. All plasmids had been diluted to 10ng/ul with 2ul of plasmid mixtures added per PCR reaction (20ng total). ‘-ve’ refers to nuclease free water template negative controls.

200ug of each plasmid arrived from GenScript at a concentration of 1ug/ul in 200ul TE buffer. Each plasmid was diluted by a factor of 100 (to 10ng/ul) and combined accounting for their size to achieve the same number of moles per plasmid within the mixture. Inserts were not separated from the plasmid backbones. Five plasmid mixtures were created: mixture 1 contained plasmids with inserts spanning amplicon targets of *dhfr*, *dhps*, *mdr1*, *kelch13*, *csp*, and three *P. falciparum* 18S rRNA sequences (representing the amplicon diversity present in the five *P. falciparum* 18S rRNA genes present in the 3D7 reference genome). The remaining four mixtures contained mixture 1, plus: 2) *P. malariae* 18S, 3) *P. ovale* 18S, 4) a mixture of *P. malariae* and *P. ovale* 18S, and 5) *P. vivax* 18S, to represent mixed-species *Plasmodium* infections *.* Serial dilutions were created for plasmid mixture 1, starting at 10ng/ul, to: 5ng/ul, 2.5ng/ul, 1ng/ul, and 0.5ng/ul. 2ul mixture 1 was added into the DRAG2 multiplex PCR assay (total reaction volume 25ul), i.e. total plasmid mixture masses added per 25ul PCR reaction were: 20ng, 10ng, 5ng, 2ng, and 1ng. From gel electrophoresis inspection, the first three concentrations (20ng, 10ng, and 5ng) produced all the expected bands with high intensity; some bands were faded at the lower concentrations. We opted to use the 20ng mixture going forwards, but would expect similar results down to at least 5ng. The plasmid cost is less than US$0.1 (10 US cents) per PCR reaction, and the 200ug starting quantity would be sufficient for at least 100,000 PCRs. Figure 1 shows the DRAG1, DRAG2-A and DRAG2-B multiplex PCR products visualised by agarose gel electrophoresis using genomic DNA and the plasmid mixtures (20ng plasmid mass).

Finally, the Oxford Nanopore Technologies (ONT) kit 14 native barcoding protocol (SQK-NBD114) also includes ‘spike DNA’, that functions as a positive control to confirm nanopore sequencing has worked for each barcode (separately from the PCRs, tested by the plasmid positive controls).

### Clinical samples

This study tested blood samples from clinical malaria patients recruited in Ghana, collected in Accra and in Navrongo in the Upper East Region. The samples were reported on previously in, ^5^ with a small number of additional samples from the same collection reported here. Patients were recruited into the study from LEKMA Hospital in Accra, and from community clinics and the War Memorial Hospital in Navrongo. Participants had symptoms compatible with malaria and tested positive for *P. falciparum* malaria by rapid diagnostic testing and microscopy. Participation required informed consent, and a detailed information sheet was provided. Samples collected included both leucodepleted venous blood (VB) and dried blood spots (DBS). Leucodepletion was performed by centrifugation and Buffy coat removal. Sample processing is described in more detail in. ^5^

To test the 18S amplicon for *Plasmodium* species identification, samples of *P. vivax*, *P. ovale* and *P. malariae* were selected based on microscopy findings. Five *P. vivax* samples were tested from patients in Laos. These samples were collected by the GenRe-Mekong study, ^19^ which performs genomic surveillance in five provinces of southern Laos. Collections were coordinated by the Center for Malariology, Parasitology, and Entomology (CMPE), Vientiane, Lao PDR, and Lao-Oxford-Mahosot Hospital-Wellcome Trust Research Unit (LOMRU), Vientiane, Lao PDR. The study is based at the Mahidol-Oxford Tropical Medicine Research Unit (MORU), Mahidol University, Bangkok. The *P. ovale* and *P. malariae* samples were collected from patients who presented with fever at two health facilities in Lomé, Togo, as part of an ongoing study of host-parasite interactions led by author LNA.

### DNA extraction

As in, ^5^ DNA extraction for the leucodepleted VB samples was undertaken using New England Labs Monarch High Molecular Weight (HMW) DNA extraction kit for cells and blood (T3050) according to the manufacturer protocol, and a subset of samples were extracted using the QIAmp DNA blood mini kit (51106) according to manufacturer instructions. DBS samples had been transferred from Ghana to the Wellcome Sanger Institute (WSI) and DNA was extracted using the QIAamp Investigator Biorobot kit on the Qiagen Biorobot Universal instrument. Further details are provided in Ref. 5.

The non- *falciparum* samples ( *P. vivax*, *P. malariae* and *P. ovale*) were DBS samples extracted using the QIAmp DNA blood mini kit (51106) according to manufacturer instructions.

In summary, while this study made use of samples that had been extracted using HMW DNA extraction kits, both the DRAG1 and DRAG2 assays also work from DNA extracted using ‘standard’ DNA extraction methods such as the QIAmp DNA blood mini kit. This is consistent with the fact that the largest amplicon fragment in either DRAG1 or DRAG2 assays is *csp*, around 1Kb in size.

We have produced an end-to-end protocol for the ‘wet lab’ components of the workflow, including details on DNA extraction, PCR, sample clean-up and ONT library preparation and sequencing, which is available in Extended data. ^27^

### PCR conditions

The two multiplex PCR reaction mixtures (DRAG2-A and DRAG2-B) are shown in Table 3, and the PCR conditions are shown in Table 4.

**Table 3.** DRAG2 PCR reaction mixtures.

DRAG2A	x1 run (μL)	DRAG2B	x1 run (μL)
5X HiFi Buffer	5	5X HiFi Buffer	5
10mM dNTP	0.75	10mM dNTP	0.75
*kelch13*- Forward	0.6	*csp*-Forward	0.75
*kelch13*- Reverse	0.6	*csp* -Reverse	0.75
*18*S -Forward	0.85	*msp*2-Foward	0.75
*18S*- Reverse	0.85	*msp2*-Reverse	0.75
*crt-C-* Forward	0.75	*mdr1-C*- Forward	0.6
*crt-C *-Reverse	0.75	*mdr1-C*-Reverse	0.6
*dhfr*-Forward	0.75	*dhps*-Forward	0.6
*dhfr*-Reverse	0.75	*dhps*-Reverse	0.6
*mdr1-N*-Forward	0.5	*crt-N *-Forward	0.6
*mdr1-N*-Reverse	0.5	*crt-N *-Reverse	0.6
Template DNA *	*	Template DNA *	*
HiFi enzyme	0.5	HiFi enzyme	0.5
Water (NFW)	Add to final vol	Water (NFW)	Add to final vol
**Total**	**25**	**Total**	**25**

* Volume of template DNA added depends on sample type (DBS, VB or plasmid control mixture). For DBS, typically we had concentrations after elution of 1-7 ng/ul, and we added 15ul per 25ul PCR reaction for both DRAG2-A and DRAG2-B. For leucodepleted VB, we had concentrations after elution of 2-500ng/ul and added 4ul sample per 25ul PCR reaction. The plasmid mixtures had been diluted to 10ng/ul and we added 2ul per 25ul PCR reaction.

**Table 4.** DRAG2 PCR thermocycler conditions.

Reaction conditions	Temp	Duration	
Initial denaturation	95°C	3 min	x35 cycles
Denaturation	98°C	20 sec	
Annealing	60-65°C	15 sec	
Extension	72°C	1 min	
Final extension	72°C	5 min	

### Library prep and nanopore sequencing

All nanopore sequencing described in this study was undertaken using ONT kit 14 chemistry with R10.4.1 flow cells using the MinION Mk1B. The native barcoding kit SQK-NBD114.24 was used for library preparation, as per manufacturer instructions. We have also tested the 96-plex native barcoding protocol using SQK-NBD114.96 as per manufacturer instructions and achieved similar results, at higher throughput and therefore reduced cost per sample. MinKNOW software (version 23.11.2 – 24.02.08) was used, running on commercially available high-performance ‘gaming’ laptop computers with an NVIDIA RTX Graphics Processing Unit (GPU) of specification 3080 or higher, as in Ref. 5. For a batch of 24 samples running on a new MinION mk1b R10.4.1 flow cell, sequencing was stopped after 14 hours. Real-time base calling was performed via the MinKNOW software interface using the dorado base caller (version 7.2.11-7.3.11) on super accuracy mode.

### Bioinformatic analysis

Fastq files produced by sequencing and real-time base calling were processed using the nano-rave Nextflow pipeline (nanopore rapid analysis and variant explorer), as in Ref. 5. Nano-rave is available open-access via GitHub at: https://github.com/sanger-pathogens/nano-rave . Briefly, nano-rave takes fastq files as input, maps the reads to reference sequences provided by the user using minimap2, ^20^ and performs variant calling with a user-selected choice of callers. In this study, the reference sequences provided were the coding sequences of genes targeted in the DRAG assays from the *P. falciparum* 3D7 reference genome, and the Clair3 variant caller was used with diploid genotypes. ^21^ An example nano-rave command line is shown below:

nextflow run main.nf --sequencing_manifest./seq_manifest_name.csv --reference_manifest./ref_manifest_name.csv --variant_caller clair3 --clair3_args “--model_path/opt/models/r941_prom_sup_g5014 --no_phasing_for_fa --include_all_ctgs” --results_dir/output_directory -with-trace

**Figure 2.**
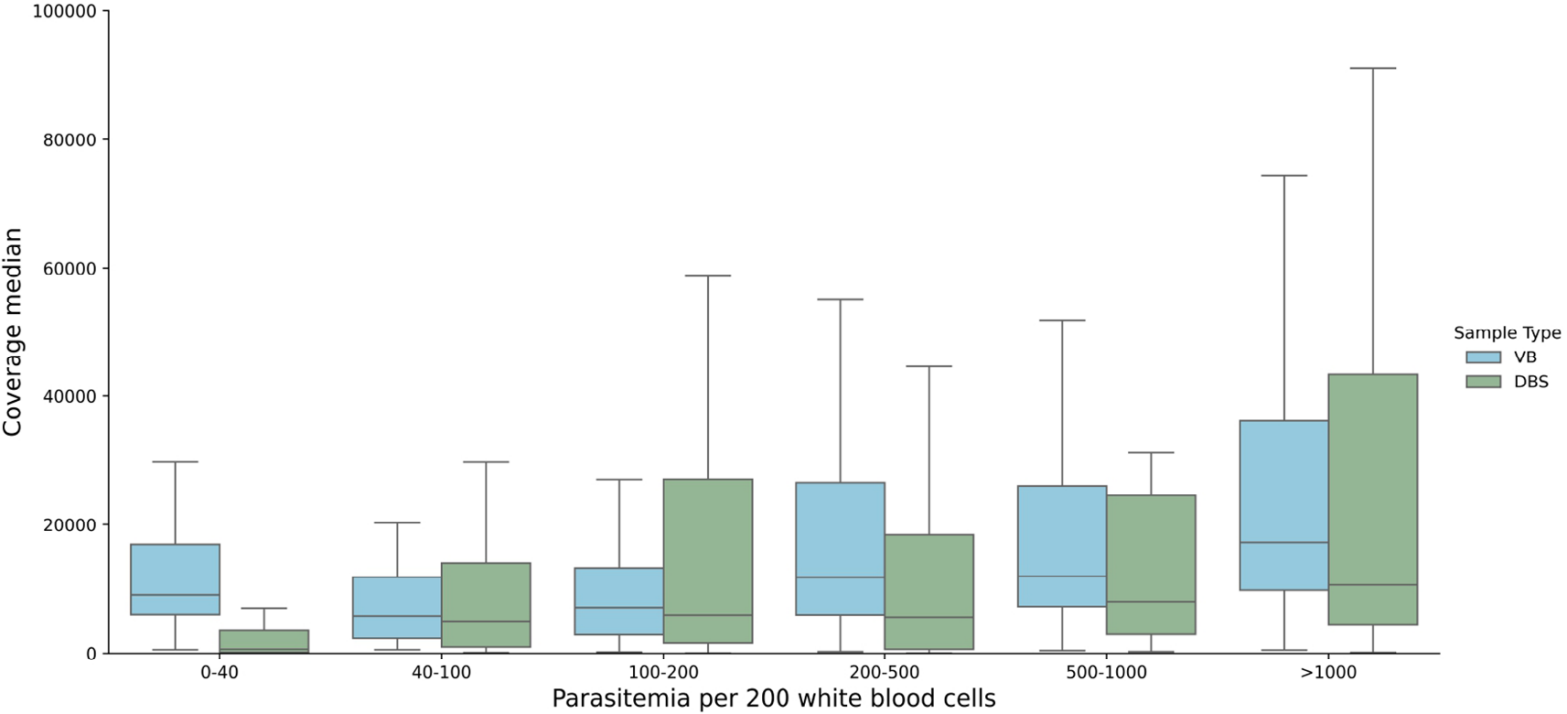
Coverage profile across all amplicons by parasitaemia.

**Figure 3.**
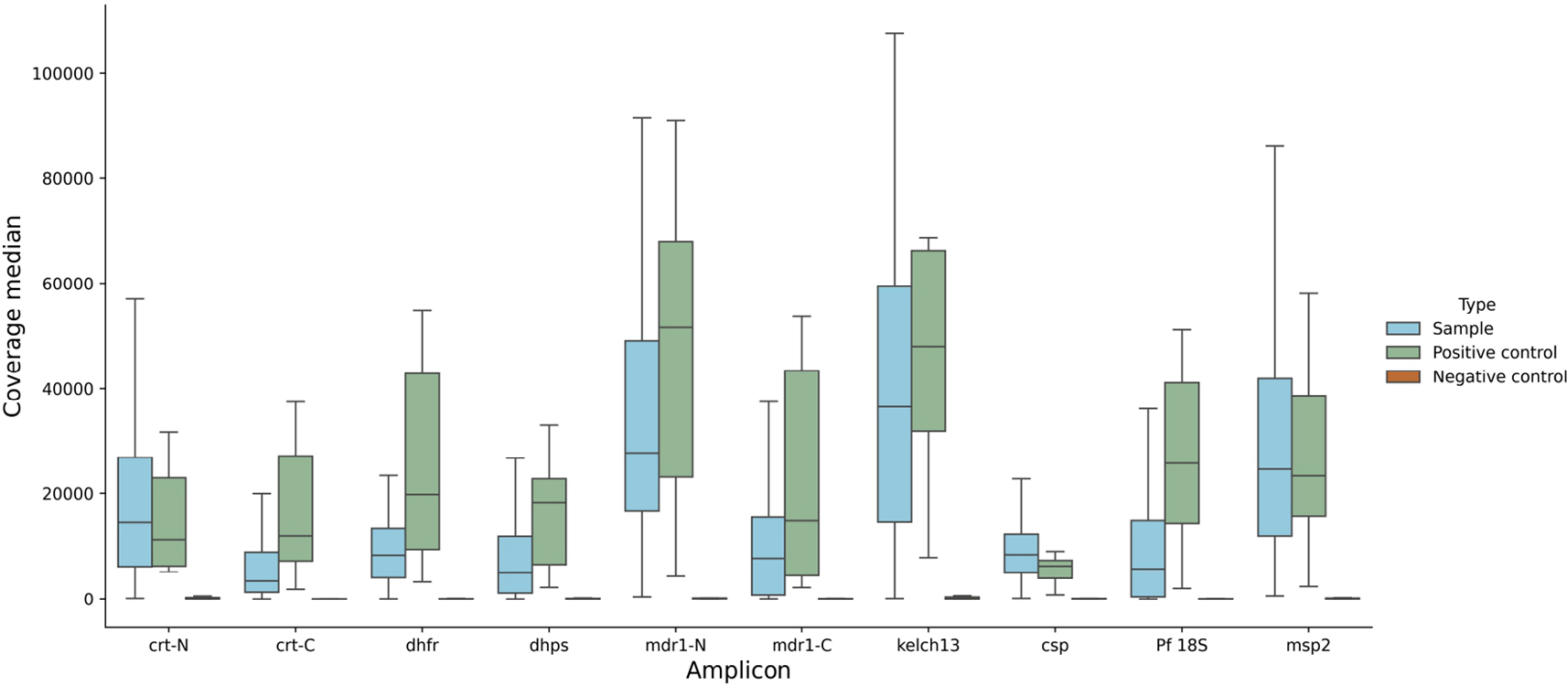
Coverage profile by amplicon. Data across all parasitaemias tested for DBS and VB samples, for positive controls, negative controls, and clinical samples. Note that coverage appears lower for *msp2*, because reads were mapped competitively against both 3D7 and DD2 *msp2* sequences, which encompass the main two allelic forms of *msp2.* The coverage shown for all amplicons depicts reads mapped to 3D7 sequences.

Nano-rave generates three output files: coverage statistics using BEDTools, variant call files (vcfs) produced by the variant caller, and quality control (QC) metrics. Coverage data for each amplicon were used to assess assay performance and are presented in Figures 2- 3. vcf files were processed using a custom R script as in Ref. 5, with the following modification: the *medaka-haploid* ^22^ variant caller was used in, ^5^ which infers haploid genotypes. We used the default diploid genotyping function of Clair3. Heterozygous genotypes (representing mixed infections) were then converted to haploid calls by using the majority allele (i.e. the allele present in >51% of reads).

For investigation of *Plasmodium* species, reads were mapped to the full-length genomes of *P. falciparum* 3D7 (Pf3D7_01_v3 version 2020-09-01), *P. malariae* (PmUG01_01_v1 version 2020-09-01), *P. vivax* (PvP01_01_v2 version 2020-09-01) and *P. ovale* (LT594505 version 2018-06-15) using minimap2. Mapped read pileups were inspected using the Integrative Genomics Viewer (IGV) software tool. ^23^ Bam files were manipulated using samtools. ^24, 25^ The Bam-readcount tool was used to calculate read counts at each position. Plots were produced using pandas, seaborn and matplotlib.

### Ethics

As in Ref. 5, ethical approval for sample collection in Ghana was granted through the PAMGEN study (ethics approval ID: NHRCIRB343, obtained from the NHRC Institutional Review Board 31/05/2019), and via the EGSAT study (ethics ID: ECBAS030/21–22, approved by the College of Basic and Applied Sciences Ethics Review Committee, University of Ghana, 20/12/2024). Ethical approval for the GenRe-Mekong study, from which the *P. vivax* samples were derived (collected in 2022 in Attapeu Province, Laos), was granted from the National Ethics Committee for Health Research (NECHR) of the Health Ministry of the Lao PDR, issued on 18 ^th^ August 2016. The Oxford Tropical Research Ethics Committee (OxTREC) approval for the study is dated 3 ^rd^ August 2016, amended 1 ^st^ August 2018. Ethical approval for the study from which the *P. ovale* and *P. malariae* samples were collected in Togo was obtained from Comité de Bioéthique pour la Recherche en Santé (CBRS), under the Ministry of Health, Public Hygiene, and Universal Access to Care (Opinion N° 045/2023/CBRS), 02/11/2023. Written informed consent was documented prior to enrolling patients into all the above studies. Approval was granted by the Wellcome Sanger Institute Research Ethics Committee and the study complied with all relevant ethical and research regulations.

## Results

### Amplicon coverage profiles

We tested the DRAG2 assay on 122 *P. falciparum* clinical samples collected from Ghana; 34 were dried blood spots (DBS) and 88 were leucodepleted venous blood (VB). 95 samples were already reported in Ref. 5, with 27 new samples from the same study collections. The samples encompassed a wide range of parasitaemias as is commonly seen in clinical collections, from 1 parasite per 200 white blood cells (WBC) to 6378 parasites per 200 WBC, or from the limit of microscopy positivity to approximately 320,000 parasites per ul of blood. Amplicon coverage for the DRAG2 assay was more even than for DRAG1. Median coverage across all DRAG2 amplicon targets, including those that failed quality control (QC) filtering, was 10,727x (interquartile range (IQR), 3,745-24,774); including only QC-pass amplicons, median coverage was 11,781x (IQR, 4938-26,925). Coverage was lower for samples in the 0-40 parasites per 200 WBC range from DBS samples ( Figure 2), suggesting that 40 parasites per 200 WBC (or approximately 2000 parasites per ul of blood, or 0.1% infected red blood cells (RBCs)) may be a pragmatic cut-off for the DRAG2 assay from DBS samples. Coverage remained high for VB samples, even at the lowest parasitaemias tested.

We analysed DRAG2 amplicon coverage for clinical samples, positive controls, and negative controls ( Figure 3). We established a pragmatic cut-off for clinical samples to be at least 7.5x the coverage of negative controls for each amplicon in the run to pass QC filters, on the basis that this would “pass” all the positive controls tested across multiple sequencing runs. We also used an absolute coverage threshold of 50x per amplicon, and if a sample has three or more amplicons that fail QC (out of the 10 amplicons in the assay) then the whole sample was failed. The poorest performing amplicon was for the *dhps* gene target, with 18 fails, whereas *csp* and *msp2* amplicons had no fails. Median coverage for each amplicon is shown in Extended data; Supplementary Table 6. ^27^ Seven samples (6%) were deemed to have failed QC on the basis of three or more failed amplicon targets, six DBS and one VB sample. These samples had low parasitaemias – the six DBS had median parasitaemia of 3 parasites per 200 WBC (range 1-27), and the VB sample had 232 parasites per 200 WBC. As a further QC step, each variant needed at least 50x coverage for valid genotyping. While coverage was fairly even over each amplicon, it declined at the C-terminal end of the *crt-C* amplicon, likely due to primer slippage in a nearby AT-rich intron.

### Drug resistance marker frequencies

Drug resistance marker frequencies were calculated from the variants called from the amplicon sequence data ( Table 5). These were consistent with the frequencies calculated from the DRAG1 assay reported in Ref. 5. No mutations associated with artemisinin partial resistance were identified in *kelch13.*

**Table 5.** Drug resistance marker counts for the DRAG2 assay. Out of 122 samples, seven were excluded due to ≥3 failed amplicons, giving N=116 samples. Individual amplicons were also excluded (QC criteria described in main text), so each amplicon has a different denominator. DRAG2 counts were tested for significance against expected proportions from the DRAG1 assay using Binomial exact test. Samples used for the DRAG1 and DRAG2 assay overlapped but with some samples unique to each assay. The SNP column shows the position of the SNP in the gene coding sequence in the 3D7 reference genome, and the base pair substitution. * *P*<0.05, **with Bonferroni correction for multiple comparisons, 0.05/11: <0.005. No frequency comparisons were statistically significantly different between the DRAG1 and DRAG2 assays.

Gene	SNP	Key Mutation (aa)	DRAG2 Frequency	DRAG1 Frequency (n=196)	*P*-value
*crt*	227 A:C	K76T	0/113	1 (0.5%)	1
*crt*	977 A:G	N326S	0/105	NA	
*crt*	1067 T:C	I356T	0/105	NA	
*dhfr*	152 A:T	N51I	90/111(81.1%)	165 (84.2%)	0.3624
*dhfr*	175 T:C	C59R	101/111(91.0%)	180 (91.8%)	0.7283
*dhfr*	323 G:A	S108N	104/111(93.7%)	183 (93.4%)	1
*dhfr*	323 G:C	S108T	0/111	0	
*dhfr*	490 A:T	I164L	0/111	0	
*dhfr*	492 A:G	I164M	0/111	0	
*dhps*	1306 T:G	S436A	57/103(55.3%)	115 (58.7%)	0.4857
*dhps*	1307 C:T	S436F	1/103 (1%)	3 (1.5%)	1
*dhps*	1310 G:C	A437G	89/103 (86.4%)	177 (90.3%)	0.1818
*dhps*	1618 A:G	K540E	0/103	0	
*dhps*	1620 A:T	K540N	0/103	0	
*dhps*	1742 C:G	A581G	2/103(1.9%)	4 (2.0%)	1
*dhps*	1837 G:T	A613S	13/103(12.6%)	27 (13.8%)	0.8862
*dhps*	1837 G:A	A613T	0/103	0	
*mdr1*	256 A:T	N86Y	4/116(3.5%)	3 (1.5%)	0.09779
*mdr1*	551 A:T	Y184F	84/116(72.4%)	139 (70.9%)	0.7601
*mdr1*	3100 A:T	S1034C	0/115	NA	
*mdr1*	3124 A:G	N1042D	0/115	NA	
*mdr1*	3736 G:T	D1246Y	0/115	NA	

### *Plasmodium* species detection

**Figure 4.**
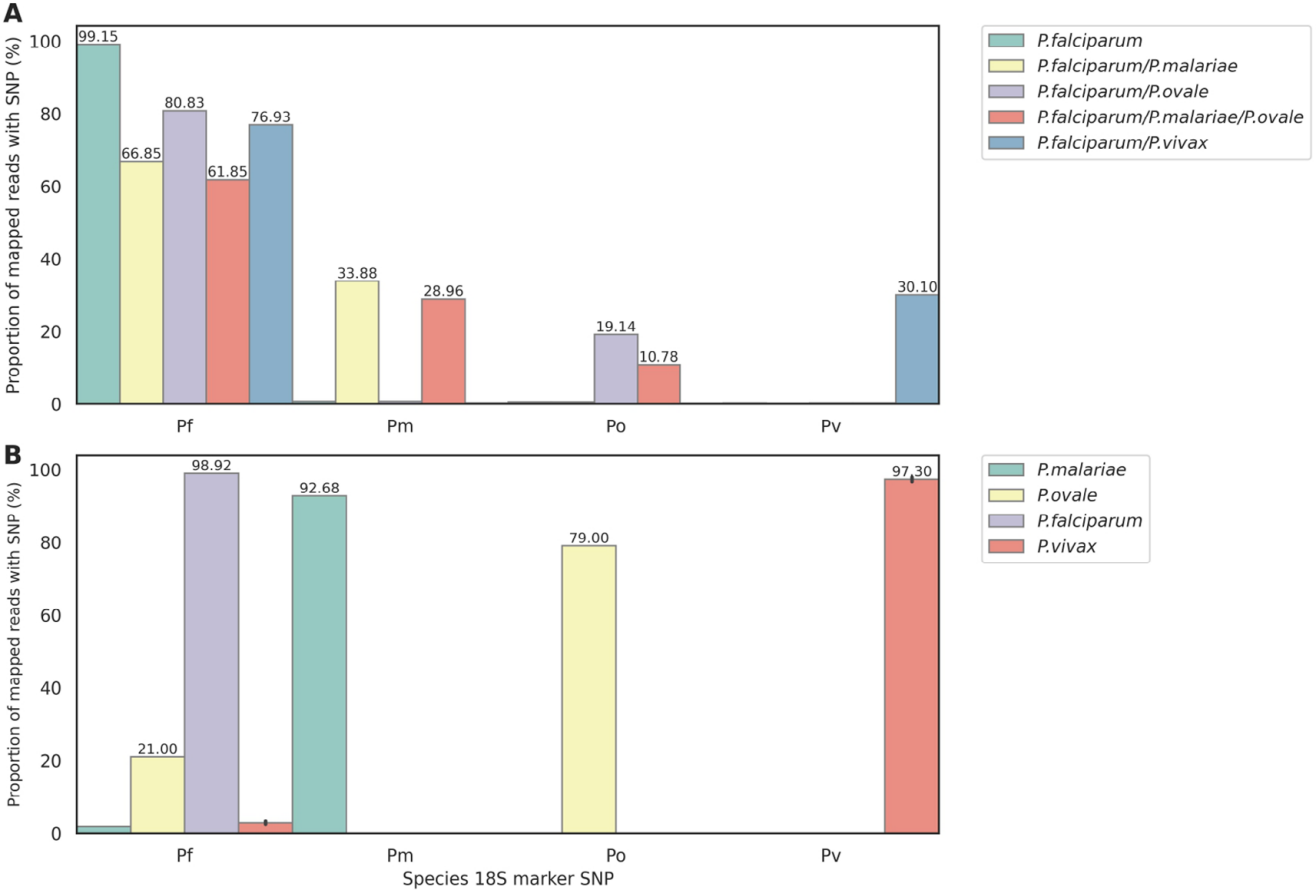
Detection of *Plasmodium* species using ‘species-determining SNPs’ from 18S rRNA amplicons. Reads were first mapped to the *Pf* 18S rRNA sequence. (B) Species-determining SNP prevalence in clinical DBS: one *P. falciparum* (Ghana), one *P. malariae* (Togo), one P *. ovale* (Togo), and five *P. vivax* (Laos).

We tested the 18S rRNA target for *Plasmodium* species detection in two ways. First, we used plasmid mixtures that included 18S rRNA sequences from *P. falciparum* ( *Pf* ), plus different combinations of *P. malariae* ( *Pm*), *P. ovale* ( *Po*), and *P. vivax* ( *Pv*) 18S rRNA sequences. We identified SNPs within the 18S rRNA sequences that were only found in their respective species, and counted the number of sequence reads containing these ‘species-determining SNPs’ Extended data; Supplementary Table 7). ^27^ As expected, for ‘pure’ *P. falciparum* plasmids, 99.15% reads mapping to the 3D7 18S rRNA sequence contained the *Pf*-determining SNPs (reflecting the accuracy of PCR and ONT sequencing). The mixtures contained approximately 2:1 ratios of *Pf* to non- *Pf* plasmids, and accordingly the proportion of reads mapping to the 3D7 rRNA gene that contained the *Pf*-determining SNPs for these mixtures ranged from 62-81%, with the vast majority of remaining reads containing SNPs corresponding to the appropriate non- *Pf* species ( Figure 4A). Next, we tested clinical DBS samples available from other studies that had been confirmed by microscopy as being *Pf* (from Ghana), *Pm* (Togo), *Po* (Togo), and *Pv* (Laos) ( Figure 4B), using only the 18S primers in singleplex reactions. Over 90% of reads mapping to the *Pf* 3D7 18S rRNA gene contained species-determining SNPs for the *Pf*, *Pm* and *Pv* samples (provided in Extended data; Supplementary Table 8)). ^27^ For the *Po* sample, 79% reads contained the *Po*-determining SNP and 21% contained a *Pf*-determining SNP; this may represent a mixed-species infection with low-level *Pf* parasitaemia. Reads were also mapped to their corresponding species reference genomes and inspected manually in IGV, and BLAST to confirm they were indeed the correct identity, matching the microscopy. These data suggest that the 18S rRNA gene target can be used as part of the DRAG2 multiplex panel to explore the prevalence of mixed *Plasmodium* species infections and the relative abundance of the non- *Pf* species from clinical samples; and also functions as a stand-alone assay for *Plasmodium* species determination.

## Discussion

This study describes a method for malaria parasite molecular surveillance using targeted nanopore sequencing and a method to use plasmids as synthetic positive and negative controls for assay performance and quality controls. We build on our previous work, ^5^ expanding the targets in the multiplex PCR to include additional regions of the drug resistance genes *crt* and *mdr1*, full-length *msp2*, and a section of the 18S rRNA gene to support *Plasmodium* species identification. We have tested the method on blood samples from clinical malaria patients over a range of parasitaemias, using both leucodepleted venous blood (VB) and dried blood spots (DBS). The method does not involve a selective whole genome amplification (sWGA) step and instead works directly from DNA extracted from clinical samples, saving time and resources. The method is effective for VB down to the lowest microscopy-positive parasitaemias, though for DBS samples the coverage drops at parasitaemias below around 40 parasites per 200 white blood cells (the same coverage threshold commonly used for sWGA ^26^). The assay can detect key drug resistance markers in the genes *crt*, *dhfr*, *dhps*, and *mdr1*; mutations in the propeller domain of *kelch13* that are associated with artemisinin partial resistance; diversity in the *csp* vaccine target and the polymorphic surface antigen *msp2* – which may be used as an approximate indicator of multiplicity of infection; and uses the 18S rRNA gene to identify *Plasmodium* species. This straightforward workflow could therefore further enhance the ability of researchers in endemic settings to provide useful data to guide national malaria control programme decision-making. The full protocol is provided in Extended data, ^27^ presented as a Standard Operating Procedure (SOP) for laboratory use.

We have used plasmids as positive control template DNA, engineering both ‘test SNPs’ and ‘control SNPs’ into the insert sequences. These plasmids can be used to confirm that the assay detects specific genetic markers of interest, such as drug resistance mutations (test SNPs), and simultaneously enable early detection of contamination of clinical samples by plasmid DNA (via the control SNPs). Because the control SNPs are extremely unlikely to occur in nature, and multiple SNPs are unlikely to arise by chance from sequencing error, contamination can be assessed on a read-by-read basis using this method, enabling absolute and relative thresholds and temporal trend data to trigger laboratory investigation for potential contamination. The plasmids are highly cost-effective, at around $0.1 (10 US cents) per PCR reaction, removing the need for gDNA from cultured parasites as positive controls. We also explored coverage cut-offs for quality control (QC) filtering, by determining the fold-changes in coverage for negative controls compared with clinical samples and plasmid positive controls. We suggest that for each amplicon, clinical samples should have at least 7.5x the coverage of the negative control to pass QC filters, in addition to an absolute threshold (for example, 50x coverage per amplicon). Another advantage is the ability to combine plasmids at precisely defined ratios, for example, to mimic mixed *Plasmodium* species infections and *P. falciparum* mixed clone infections. These features make plasmids well suited to assay quality assurance processes.

This study has several limitations. The DRAG2 assay has been tested using a previously collected sample set from Ghana ^5^ and a small number of samples with non- *falciparum* malaria species, which did not include *P. knowlesi.* The assay should be implemented as a practical workflow in laboratories based in malaria endemic countries to assess ‘real world’ performance on a larger sample size.

In conclusion, we have developed an expanded version of our previous multiplex assay for malaria molecular surveillance (MMS) using targeted nanopore sequencing; we provide assay validation data over a range of clinical sample types and parasitaemias, and described the use of engineered plasmid vectors for use as controls. A detailed SOP is provided in Extended data, ^27^ intended for use by laboratories adopting this workflow for MMS. Protocol standardisation, use of positive and negative controls, and systems of internal and external quality assurance (IQA and EQA) are essential components of assay quality assurance, which must be developed for malaria molecular surveillance to transition from a research tool to clinical and public health applications.

## Author contributions

**Table:** 

Author name	Contributions (CRediT)
Alexandria J. R. Harrott	Data Curation, Formal Analysis, Investigation, Methodology, Validation, Visualization, Writing – Review & Editing
Collins M. Morang'a	Data Curation, Formal Analysis, Investigation, Methodology, Project Administration, Writing – Review & Editing
Richard D. Pearson	Formal Analysis, Supervision
Mona-Liza Sakyi	Investigation
Ahmed Osumanu	Investigation
Enock K. Amoako	Investigation
Fagdéba David Bara	Investigation
Myra Hosmillo	Investigation, Methodology, Project Administration
Kess Rowe	Investigation, Project Administration
Yaw Aniweh	Investigation, Supervision
Gordon A. Awandare	Funding Acquisition
Francis Zeukeng	Funding Acquisition, Supervision
Ian Goodfellow	Funding Acquisition, Methodology, Supervision, Writing – Review & Editing
Cristina V. Ariani	Funding Acquisition, Supervision
Lucas N. Amenga-Etego	Conceptualization, Data Curation, Formal Analysis, Funding Acquisition, Investigation, Methodology, Project Administration, Supervision, Validation, Visualization, Writing – Review & Editing
William L. Hamilton	Conceptualization, Data Curation, Formal Analysis, Funding Acquisition, Investigation, Methodology, Project Administration, Supervision, Validation, Visualization, Writing – Original Draft Preparation, Writing – Review & Editing

## Data Availability

Nanopore sequence data for all samples analysed in this study is available via the European Nucleotide Archive (ENA), with sample codes provided in Supplementary Table 9. Prior to upload, reads were mapped to the *Plasmodium falciparum* 3D7 v3.0 reference genome and only mapped reads were retained, to filter out human reads. Supplementary materials relating to this paper can be found here: Figshare: An expanded method for malaria parasite genetic surveillance using targeted nanopore sequencing. https://doi.org/10.6084/m9.figshare.28539320.v1. ^27^ The project contains the following underlying data:
1) Supplementary Tables (1-9) 2) Plasmid mix SOP.pdf 3) DRAG lab SOP.pdf 4)
plasmid_insert_seqs.zip Data are available under the terms of the Creative Commons Attribution 4.0 International license (CC-BY 4.0).
